# Bayesian-Based Hyperparameter Optimization of 1D-CNN for Structural Anomaly Detection

**DOI:** 10.3390/s23115058

**Published:** 2023-05-25

**Authors:** Xiaofei Li, Hainan Guo, Langxing Xu, Zezheng Xing

**Affiliations:** 1 College of Transportation Engineering, Dalian Maritime University, Dalian 116026, China; guohainan@dlmu.edu.cn (H.G.); xlx@dlmu.edu.cn (L.X.); 2 College of Information Science and Engineering, University of Jinan, Jinan 250022, China; xing_zz@stu.ujn.edu.cn

**Keywords:** structural anomaly detection, 1-D convolutional neural network, Bayesian optimization algorithm, decision-level fusion, vibration signals

## Abstract

With the rapid development of sensor technology, structural health monitoring data have tended to become more massive. Deep learning has advantages when handling big data, and has therefore been widely researched for diagnosing structural anomalies. However, for the diagnosis of different structural abnormalities, the model hyperparameters need to be adjusted according to different application scenarios, which is a complicated process. In this paper, a new strategy for building and optimizing 1D-CNN models is proposed that is suitable for diagnosing damage to different types of structure. This strategy involves optimizing hyperparameters with the Bayesian algorithm and improving model recognition accuracy using data fusion technology. Under the condition of sparse sensor measurement points, the entire structure is monitored, and the high-precision diagnosis of structural damage is performed. This method improves the applicability of the model to different structure detection scenarios, and avoids the shortcomings of traditional hyperparameter adjustment methods based on experience and subjectivity. In preliminary research on the simply supported beam test case, the efficient and accurate identification of parameter changes in small local elements was achieved. Furthermore, publicly available structural datasets were utilized to verify the robustness of the method, and a high identification accuracy rate of 99.85% was achieved. Compared with other methods described in the literature, this strategy shows significant advantages in terms of sensor occupancy rate, computational cost, and identification accuracy.

## 1. Introduction

Accelerometers are one of the most technologically mature sensors, and are widely used in various industries and fields [1,2]. In the field of Structural Health Monitoring (SHM), acceleration data are one of the key responses monitored [2], and can be used to characterize the vibration characteristics of a structure. The parameters of structural dynamic characteristics, such as vibration patterns, frequencies, damping ratios, etc., can be obtained from large amounts of acceleration monitoring data [3]. Under the long-term influence of the environment and load effects, structures may have problems such as material weakening and component damage, leading to changes of parameters in the local structure (such as stiffness and mass), which will alter the global dynamic characteristics of the structure [4]. The sensitivity of acceleration data to structural parameter changes has attracted significant academic interest, particularly in the study of vibration-based methods for identifying structural damage [5].

Research into vibration-based SHM technology has been performed with the aim of identifying the existence, location and degree of damage to a structure [6]. These techniques can be broadly categorized into two major kinds of identification method: parameter-based methods and non-parameter-based methods. In traditional identification parameter-based methods, specific feature parameters are extracted from measured acceleration data, such as the natural frequency [7], vibration type [8,9,10], vibration curvature [11], and frequency response function [12,13,14,15]. Then, structural damage is evaluated by a variety of ways, such as calculating damage indicators, setting thresholds, or the mapping relationship between feature information and structural damage was established with the use of machine learning methods [16,17,18,19,20]. Research has demonstrated the good damage identification ability of these methods; however, these methods do have some shortcomings, such as the heavy reliance on the expertise and knowledge of specialists, the need for manual feature extraction, their low level of automation, and their unsuitability for performing real-time analysis of massive amounts of monitoring data. With the improvements in technology and the reduction in the price of sensors, the amount of acceleration data that can be collected has increased; therefore, the accurate and efficient extraction of damage features from massive datasets, along with the evaluation of the damage itself, has become a significant challenge in contemporary times [21].

Emerging deep learning methods are providing new ideas for the efficient processing of big data, especially in the case of convolutional neural networks (CNN), which demonstrate excellent ability in image classification and identification, target positioning and other fields [22]. CNNs have garnered significant attention in the fields of structural health monitoring and structural damage diagnosis. In the second section, structural damage identification methods based on CNN are described in detail. Among them, 1D convolutional neural network (1D-CNN) can directly process 1D vibration signal data. High levels of detection accuracy can be achieved with small amounts of training data. This has potential for use in structural damage identification. The basic principle is to realize damage identification by establishing the mapping relationship between characteristic parameters and the structural damage state. Large amounts of monitoring data can be processed efficiently and accurately by 1D-CNN. In recent years, 1D-CNN has played an important role in research related to health monitoring technology for use in important engineering structures, such as bridges, high-rise buildings, offshore wind turbines, high-pile docks, steel frame structures, etc. However, for different structural damage identification application scenarios, the proposed model network architecture and its parameters will be different, and the optimal identification accuracy will only be achieved for specific research objects. Therefore, whether or not 1D-CNN can successfully solve specific problems, especially for structural damage detection problems, depends on finding the appropriate network architecture and parameters. There are many cutting-edge optimization algorithms for parameter optimization [23,24], including grid search, random search, Bayesian optimization, etc. Of these, Bayesian optimization has been applied in many fields and has achieved good results. João et al. [25] studied the effects of five strategies, namely greedy, random, Bayesian, hyperband, and genetic methods, on the search process of the 1D-CNN architecture using a real clinical case. Vigneshwaran et al. [26] proposed using a CNN model optimized by the Bayesian optimization algorithm to identify the state of 11 kV high-voltage (HV) polymer insulators. Mohamed et al. [27] processed magnetic resonance images using a CNN model to identify brain tumor types. Bayesian optimization was used to search for the optimal hyperparameters of the CNN model. Shen et al. [28] proposed a multi-wavelength SpO_2_ measurement method based on a 1D-CNN model. On the basis of a comparison of the two hyperparameter optimization algorithms, Bayesian optimization was found to be superior to Grid Search CV. Tang et al. [29] built an adaptive CNN model based on Bayesian optimization. Intelligent and accurate diagnosis of hydraulic pump faults was realized. These studies demonstrated the excellent ability of Bayesian optimization to perform hyperparameter optimization. However, there are currently few studies on the optimization of 1D-CNN hyperparameters for structural damage identification problems. The usual method is to adjust the structure and configure the parameters on the basis of the existing network structure. Currently, most hyperparametric tuning approaches are based on empirical knowledge and manual testing, but this process is a challenging, complex, and time-consuming process that requires adequate expertise, experience, and testing.

Therefore, in this paper, a strategy is proposed for building a 1D-CNN model that is suitable for diagnosing damage in different types of structures. This strategy first establishes a general network architecture that integrates the 1D-CNN feature extraction module and the multi-layer perceptron decision module. On the basis of multiple tests, the range of hyperparameters in the model is determined. In the model optimization process, the Bayesian optimization algorithm is embedded in the network architecture to adaptively select the relatively optimal hyperparameter combination. After optimization, the model is combined with data fusion technology, which greatly improves the robustness of the deep learning model in structural damage identification applications. This work was carried out and validated on a self-designed simply supported beam model and a framework benchmark model publicly released by a research institution. By using a public dataset to perform this research, the advantages of the proposed method in this paper can be highlighted on the basis of a comparison with research work in the existing literature.

This paper is organized as follows: Section 2 describes the related works. Section 3 describes the method used in this study in detail, including the data processing method and the 1D-CNN model architecture; Section 4 presents a preliminary study utilizing a custom-built simply supported beam test model, and delves into the effectiveness of the proposed strategy; in Section 5, the accuracy and robustness of the proposed method are verified by means of structural health monitoring benchmark tests; in Section 6, the sensitivity analysis and scalability analysis of the proposed method are discussed in detail; and finally, in Section 7 the main conclusions are drawn and potential directions for future research are suggested.

## 2. Related Work

The method of using CNN for structural damage identification has been widely studied. In previous research, some attempts have been made to convert one-dimensional signals into two-dimensional maps, including the use of recurrence maps [30,31], continuous wavelet transform [32], and Hilbert–Huang transform [33], and through the CNN model, the mapping relationship between the two-dimensional maps and the structural damage was established. During the investigation, it was found that these methods have some flaws, such as high computation cost, considerable hardware requirements, and demand for high quantities of training samples, as well as the complex process for parameter selection during the data dimension transformation, which is not suitable for real-time analysis of a large amount of monitoring data.

Compared to two-dimensional convolutional neural networks, one-dimensional time series data can be used directly for training with 1D-CNN, achieving high levels of detection accuracy with a small amount of training data. In recent years, related research has shown its excellent ability in the field of structural health monitoring. Abdeljaber et al. [34] utilized 1D-CNN to identify the damage of a laboratory frame structure at Qatar University [35], accurately detecting node bolt loosening and joint damage. Building upon this, they proposed a method [36] for evaluating the overall damage level of frame structures, using experimental data published by the International Association for Structural Control (IASC)-American Society of Civil Engineers (ASCE) [37] and training a 1D-CNN model with a dataset to approximate different damage states within two extreme state ranges. Lin et al. [38] used a 1D-CNN to identify single or multiple instances of damage in a simply supported beam. The beam model consisted of 10 equally spaced Euler–Bernoulli beam elements, and the acceleration signals of the internal nine node positions were collected as input data. Zhang et al. [39] proposed a 1D-CNN method that was used to identify the local stiffness and mass changes in the structure. Sharma et al. [40] used a vibration response at the midpoint of the beam element to train the 1D-CNN network, and identified the damage at the two nodes of the element. This finding can reduce the number of sensors for structural monitoring. Zhou et al. [41] applied the 1D-CNN model to the problem of identifying the structural damage of high-pile docks. The results show that 1D-CNN can directly extract damage-sensitive features from displacement response data. Almutairi et al. [42] used 1D-CNN to evaluate the damage degree of the cantilever, and the influence of different excitation forms and the number of sensors on the accuracy of identifying the damage was analyzed. Teng et al. [43] used 1D-CNN to detect damage in bridges, and used the decision-level fusion strategy to combine the prediction results from multiple CNNs, which significantly improved the accuracy. Flah et al. [44] used 1D-CNN for damage identification in mid-high-rise building structures; it was proposed that installing a sensor every six floors may achieve good identification results. This method can reduce the number of sensors used for monitoring mid-high-rise buildings, which may be more cost effective and practical.

1D-CNN has shown good ability to perform damage diagnosis in different structures. In the above literature, in the application scenarios of different structures, the model architecture and hyperparameters of 1D-CNN were specific. Due to the different monitoring networks of different structures and different signal sampling frequencies, the proposed model only shows strong sensitivity to datasets obtained from specific structures. In confronting new structural damage identification problems, the 1D-CNN model and hyperparameters need to be adjusted according to the actual situation, which is a very complicated process. It is worth noting that in the above research, Bayesian optimization technology was not applied to the automatic selection of optimal hyperparameters of the 1D-CNN model in the field of structural damage identification. In addition, in this paper, the use of less sparse sensor data as damage assessment indicators is considered to achieve high-precision damage detection of all local locations of the overall structure, which also has considerable reference value for the study of structural health monitoring technology.

## 3. Materials and Methods

**Figure 1:**
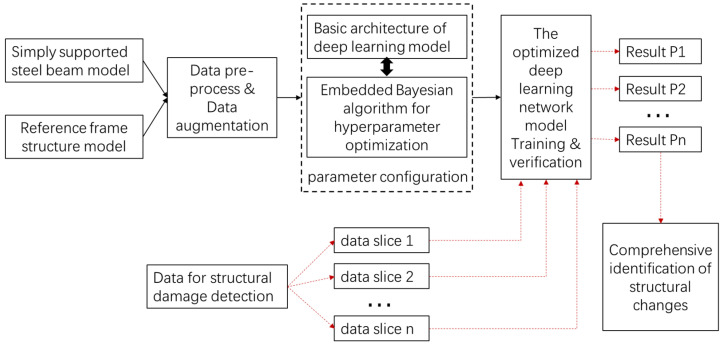
Overview of the methodology.

In this section, the organizational framework of the proposed strategy is described, as shown in Figure 1, which outlines the research methodology of this paper, and which will be described in detail in the following subsections.

### 3.1. Dataset Pre-Processing

**Figure 2:**
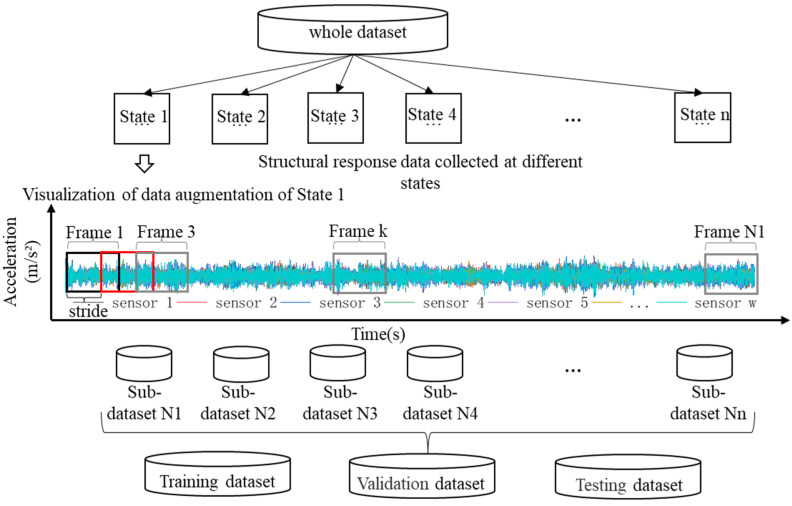
Dataset pre-processing methods.

In the experimental process, structural response data were collected under n different states through a signal acquisition system. The number of sensors was *w*, the sampling time was *L* s, and the sampling frequency was *f* Hz. In the training process of the 1D-CNN model, there are requirements regarding the size of the input data, whereby independent data samples of the same size as a certain batch are needed, rather than a complete continuous time series. Therefore, in this paper, the sliding window method was used to augment the dataset. Sliding window is a commonly used way of decomposing signal data, and the method used to divide the dataset is shown in Figure 2, that is, a window with a size of *h* × *w* (where the window length is *h* in the time dimension, and the window width is *w* in the sensor dimension) is selected, dividing the whole dataset into *N* identical sub-data samples, with a time step of *s*. When the quantity of data samples is small, data augmentation can be achieved by overlapping adjacent two windows. The total number of samples *N* can be calculated using Equation (1):

(1) N=1s×Lf−h+1

To increase the convergence speed and generalization ability of the trained model, it is necessary to normalize the data before decomposition, as shown in Equation (2).

(2) $$\hat{L}=\frac{L_{0}-\bar{L_{0}}}{\sigma (L_{0})}$$

where $L_{0}$ is the original dataset, $\bar{L_{0}}$ and $\sigma (L_{0})$ are the mean and standard deviation of the original data, respectively, and $\hat{L}$ is the normalized dataset.

### 3.2. Basic Architecture of the Deep Learning Model

**Figure 3:**
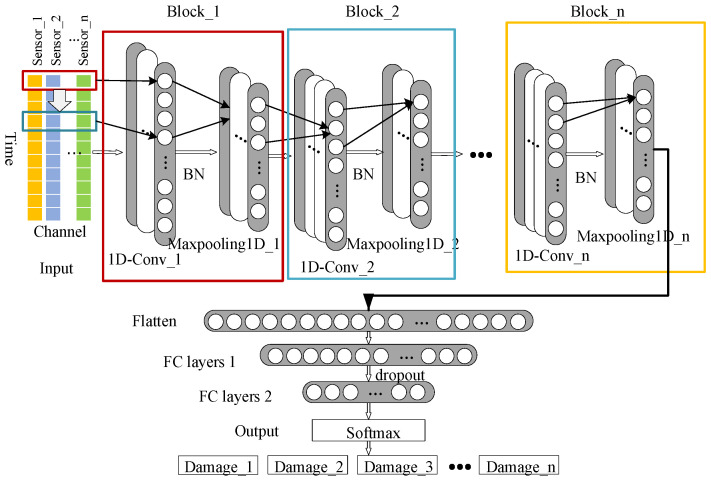
1D-CNN basic architecture.

This paper proposes a general model architecture for structural damage diagnosis. This model architecture consists of a convolutional neural network module and a multi-layer perceptron module. The former is mainly used to extract feature data that better represents damage from structural acceleration data, while the latter is mainly used to establish a mapping relationship between feature data and damage types, enabling structural damage diagnosis functions. Figure 3 shows the basic architecture of the proposed deep learning model. The theoretical and functional aspects of each layer are described in detail in the following subsections.

#### 3.2.1. One-Dimensional Convolutional and Pooling Layers

The one-dimensional convolutional layer and pooling layer are the core functional layers of the feature extraction module, and they are used to extract feature maps from the normalized acceleration data samples. Each one-dimensional convolutional layer contains *K* neurons, which perform filtering on the input data; the mathematical equation for this is shown as Equation (3).

(3) $$h_{k}=f\left(conv1D\left(w_{k},X\right)+b_{k}\right)$$

(4) $$conv1D\left(w_{k},X\right)\left[i\right]=w_{k}⊗{\left[x_{i},x_{i+1},\cdots ,x_{i+h}\right]}^{T}$$

where *X* is the input data of the convolution layer, and its input dimensions are (*height*, *width*, 1), where the width value is equal to the number of sensors and the height value is equal to the number of data contained in time dimension; *w_k_* is the convolution kernel weight vector of the convolution layer, with dimensions of (*KSIZE*, *width*), where *width* is equal to the width of the input data sample, so *KSIZE* is the main hyperparameter determining the size of the convolution kernel; *conv*1*D*(…) is a one-dimensional convolutional operator that is calculated by moving the convolution kernel only in the direction of the height of the data sample for the convolution calculation, as shown in Equation (4) for the *i*-th step of the convolution calculation process. This is the main difference between 1D-CNN and the 2D-CNN, which has the characteristics of compact computation, and has low demand for computational resources, and is therefore suitable for processing real-time monitoring data using low-cost hardware. *f* is the nonlinear activation function. After the filtering effect of the convolutional layer, multiple feature maps are outputted. Among them, many feature maps will contain some useless feature information. Therefore, a pooling layer is connected after the convolutional layer. The pooling function is achieved by computing the maximum value in a specific area or taking an average of the values in a specific area. The pooling operation emphasizes certain features without changing the characteristics of the feature maps, which increases the robustness of the model. Meanwhile, it can also compress the dimensions of the feature maps, and as the stride of the sliding window increases, the dimensions of the feature maps decrease.

#### 3.2.2. Fully Connected Layer

The last few layers of the fully connected layers form the multi-layer perceptron decision maker for structural damage identification. The main function of these layers is to establish a mapping relationship between the feature maps extracted by the feature extraction module and the types of structural damage. The mathematical formula is shown in Equation (5).

(5) Y=f(∑h×w+b)

where *h* is the output feature of the convolutional layer feature extractor, *w* and *b* denote the weights and additional bias terms, *f* denotes the activation function, and *Y* denotes the output of the fully connected layer. The last fully connected layer is the output layer. In this layer, the SoftMax function is often used for classification problems. For regression problems, the linear function can be used; the remaining fully connected layers are implicit layers, and all use the ReLU activation function.

#### 3.2.3. Batch Normalization (BN) Layer and Dropout Layer

Several tricks are applied to improve its generalization capabilities. These techniques include the BN layer and the dropout layer. During the training of a deep learning model, as the feature extraction layer is learned in depth, instabilities in the backpropagation operation may occur, such as gradient disappearance or gradient explosion. Batch normalization is a method for preventing “internal covariance bias”. The mean and variance of each batch of training data are calculated, and then the original data is shifted and scaled to the new scale data, whose mean is zero and variance is 1. This method of reparameterization using BN helps to alleviate the problem of coordinated updates among the layers in the neural network. Better results can be obtained by normalizing the mean and standard deviation of each feature and creating new values using scale factors and offset factors.

(6) $${\hat{h}}_{i}=\frac{h_{i}-\mu_{B}}{\sqrt{\sigma_{B}^{2}+\varepsilon }}$$

(7) $$x_{i}=\gamma \hat{h_{i}}+\beta \equiv BN_{\gamma ,\beta }\left(h_{i}\right)$$

where *µ_B_* denotes the mean value of small batch *B* and $\sigma_{B}$ denotes the standard deviation of small batch *B*. The parameters *γ* and *β* are learned by backpropagation, which adjusts the normalized values to avoid being driven to zero. The parameter *ε* is a very small constant used to prevent the denominator from becoming zero.

Dropout is a technique for solving the problem of overfitting in neural networks by reducing the coadaptation between neurons to generate more efficient training. If the loss occurring during the training process is significantly smaller than the loss occurring during the testing process, then an overfitting situation can occur. The core idea is to randomly disconnect the connections between neurons with a fixed specific disconnection ratio. This technique not only mitigates the overfitting problem in neural networks but also improves the generalization ability of the model. It is worth noting that the introduction of dropout does not change the structure or parameters of the model, it simply randomly drops the output of some neurons during the training process. Special attention is needed when choosing an appropriate disconnection ratio, which should normally be between 0.2 and 0.5. An improperly chosen disconnection ratio may lead to underfitting or overfitting problems. Therefore, to obtain the best performance, the parameters of dropout can be optimized as hyperparameters.

### 3.3. Bayesian Optimization of Hyperparameters

Hyperparameter optimization is a key part of the new structural damage identification strategy proposed in this paper. For different structural damage diagnosis problems, the response datasets of different structures belong to multi-source domain datasets, and there are significant differences. No model is able to solve all structural damage identification problems, even for models that perform exceptionally well for specific problems. When 1D-CNN is applied to specific structural damage identification problems, the primary challenge is to reconstruct the network structure and configure the hyperparameters, or to adjust them on the basis of excellent existing network structures. Both processes require a rich body of experience and professional knowledge, and are undoubtedly difficult. Therefore, in this paper, a trick algorithm for performing hyperparametric optimization, namely Bayesian optimization, is used [45]. In previous studies, it has been shown that Bayesian optimization algorithms are more suitable for deep learning hyperparameter optimization than optimization algorithms such as network search and random search [46], and Bayesian optimization can find the optimal hyperparameters efficiently and with fewer iterations [47].

The basic steps of the Bayesian optimization algorithm are shown in Algorithm 1. According to the basic structure of the convolutional neural network, a set of *n* hyperparameter elements, including structural and training hyperparameters, are determined to form the set *x*. Structural hyperparameters include the number of convolutional layers, the number of filters in each convolutional layer, kernel size, stride, the number of pooling layers, pooling kernel size, padding, the number of fully connected layers, the number of neural units, BN layers, dropout layer parameters, and the activation function. The training hyperparameters are the tuning optimizer, learning rate, batch size, and the ratio of the training set to the test set. The search range for all hyperparameters is determined with reference to related research experience and test experiments. The initialized parameter set *D* is obtained by training the model initialization, and it is assumed that the parameter set satisfies the proxy model *M*. Based on the prior distribution and the known parameter set *D*, the posterior distribution *p(y|x*, *D)* is calculated, and the acquisition function *S* is constructed using the posterior distribution. By using the acquisition function, the next set of hyperparameters *x_i_* is selected to be trained in the network, and the output *y_i_* of network model evaluation value is obtained. After *T* iterations, an approximately optimal combination of hyperparameters is obtained.
**Algorithm 1: Bayesian Optimization Hyperparameter Framework**$\begin{array}{l} Input:\;The\;number\;ofparameters\;n_{0},\;The\;maximum\;number\;of\;iterations\;T,\\ \;The\;search\;space\;for\;parameters\;X,\;The\;function\;for\;evaluate\;model\;f,\\ \;Surrogate\;models\;M,\;Acquisition\;Function\;S \end{array}$Output: The optimal parameter combination xD←InitSamples(f,X)$For\;i\leftarrow {\left|D\right|}_{}to\;T_{}\;do:$$\;\;p\left(y|x,D\right)\leftarrow FitModel(M,D)$$\;\;x_{i}\leftarrow \underset{x\in X}{\arg\max}S\left(x,p\left(y|x,D\right)\right)$$\;\;y_{i}\leftarrow f(x_{i})$$\;\;D\leftarrow D\cup \left(x_{i},y_{i}\right)$end for

To determine the search range for each hyperparameter element, the search range for each hyperparameter was specified with reference to different domain studies and conducting several experiments, and the specific values are shown in Table 1.

### 3.4. Structural Local State Change Identification

In the previous subsection, the basic structure of the proposed 1D-CNN and the specific functions of each structural layer were described, and the method for embedding a Bayesian optimization algorithm into the network structure in order to obtain optimal hyperparameter combinations was described, which can be used to obtain a high-performance 1D-CNN model that is able to recognize structural damage. The application of the method to the structural damage identification problem consists of three phases: the parameter optimization phase, the training phase, and the identification phase. In the training phase, a training dataset, which is a dataset of correspondences between acceleration sensor data samples and structural damage types, needs to be established. The acceleration data samples can be obtained using the data preprocessing method described in Section 3.1, while the structural damage types need to be encoded with labels for easy reading in the model. The labels are designed as a vector consisting of *n* elements (*n* is equal to the element numbers of all monitored locations of the structure). Each element value indicates the degree of damage at the monitored location. All of the acceleration data samples and label codes constitute the dataset for training the network model. The acceleration data samples are fed into the network model, the predicted label codes for the damage type are output, and the loss function is used to estimate the difference between the predicted and true label codes, and to update the neural network according to their gradient values. The parameter optimization phase consists of several model training stages, and the final 1D-CNN network structure and configuration parameters for a given structural damage identification problem are established by the Bayesian optimization of hyperparameters in Section 3.4; in the next step, this optimal network structure is trained using the dataset, and the coefficient weights in the network are updated to preserve the optimal model as a classifier for the damage identification problem.

**Figure 4:**
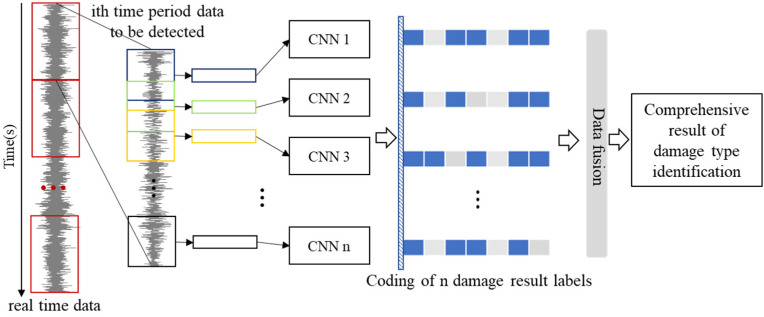
1D-CNN combined with the data fusion technology identification method.

In the identification phase, the new response data of the structure are collected, and the damage type is predicted using the trained model. However, even very good network models are not able to identify the damage occurring in a structure completely and accurately. All of them have certain defects as a result of the influence of environmental disturbances, sensor instability or other factors. Some basic characteristics of vibration signals can be easily changed, causing misjudgment of structural damage. Therefore, on the basis of the optimized 1D-CNN model, data fusion technology is used to build a comprehensive decision model for decision-level fusion [48]. The decision-level fusion proposed in this paper means that a certain damage identification method is used to process and identify multiple data fragments within a certain period of time, and the obtained damage identification results will be decision fused. Comprehensive damage identification information is extracted to make the highest-level decision. The data fusion technique is applied as shown in Figure 4, where the data in a fixed time period are collected continuously by a dynamic signal acquisition system in order to identify the structural damage types, and the sampling frequency is set to *f* Hz. The individual *T* s data are enhanced into *n* data samples using the sliding window slicing method described in Section 3.1, and these samples are then used as the input of *n* 1D-CNN recognizers, and *n* damage label codes are output. These identification results are the results of structural state evaluation in the same time period by default, ignoring structural state changes in short time periods. Then, the final structural damage diagnosis results are obtained using the data fusion calculation method to synthesize *n* damage label coding information results. Two data fusion calculation methods, namely the mean value method and the voting method, are considered. When only structural damage location identification is considered, the problem is one of multi-task classification identification. The voting method can calculate comprehensive results efficiently and stably. When the location and degree of structural damage need to be identified, the task corresponds to solving the regression problem, and the mean value method is more suitable for performing the data fusion of the output layer results.

The network architecture design was based on the Tensorflow framework and Keras API in the Python 3.7 language environment, with an Intel^®^ Xeon^®^ Gold 5218R CPU and an NVIDIA Quadro RTX 5000 graphics card to accelerate the training.

## 4. Case Study 1: Identification of Structural Parameter Variations of Simply Supported Beams

### 4.1. Experimental Design for Investigation of Simply Supported Beams

**Figure 5:**
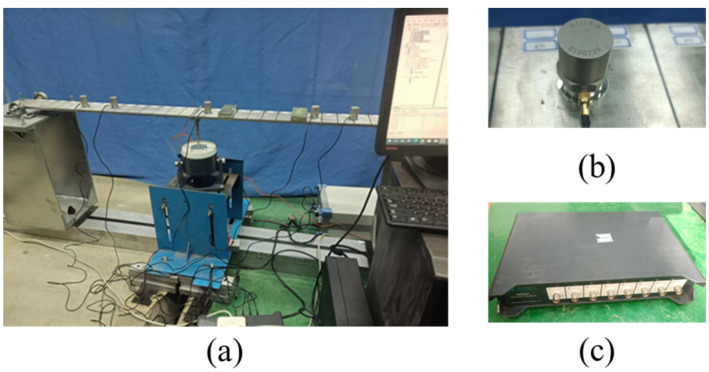
Simply supported beam test. (**a**) Simply supported beam model; (**b**) acceleration sensing network; (**c**) dynamic data collector.

As a preliminary study, a stainless steel simply supported beam with a length of 1.5 m was considered, which had a rectangular cross-section with a width of 0.1 m and a height of 0.02 m. It was divided into 30 equal beam elements, as shown in Figure 5a. The material properties were as follows: Young’s modulus of 206 GPa and density of 7900 kg/m^3^. A shaker was used as the excitation source of the external load, the random loads were generated by controlling the exciters through a signal generator to simulate environmental loads.

**Figure 6:**
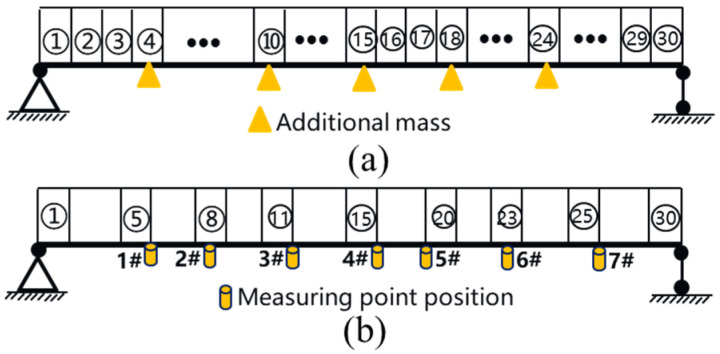
Schematic diagram of a simply supported beam. (**a**) Additional mass location; (**b**) measurement point arrangement.

For structural health monitoring research and application, sensor arrangement is a very important aspect, and a reasonable arrangement form is better able to characterize the overall characteristics of the structure. In [38], it was shown that the features extracted by the 1D-CNN model from acceleration data are mainly the inherent frequency and vibration mode of the structure, so this study considers the arrangement of acceleration sensors at the position corresponding to the peak of each order of vibration mode and compares it with the other three arrangement forms, which are analyzed in Section 6. In this case, seven AI050 acceleration sensors (see Figure 5b) were arranged on the beam, with specific positions as shown in Figure 6b. The acceleration data were collected using a JM5937 dynamic data acquisition instrument (see Figure 5c), data line and dynamic acquisition analysis software, and other dynamic signal acquisition systems.

In order to verify the performance of the proposed method, it is necessary to obtain acceleration data samples corresponding to the healthy and damaged states of the structure. In this study, additional mass blocks on the beam structure were used to increase the local structural stiffness and mass of the simply supported beam in order to simulate structural damage [39]. The sensitivity of the proposed method to small local variations in the stiffness and mass of the actual structure can then be verified. One or more elements were randomly selected for mass adjustment on the beam structure, and some stainless steel plates were used as additional masses in this experiment, as shown in Figure 6. Each mass block had dimensions of 100 mm × 50 mm × 5 mm and weighed about 196 g. The degree of damage depends on the number of plates attached to the beam. Three degree levels were set using three plates. These additional masses were 1.45% (Level 1), 2.90% (Level 2), 4.35% (Level 3) of the beam mass, respectively. The scenario of structural single-position mass change and the scenario of structural multi-position mass change were considered, and five elements were randomly selected in different areas of the beam structure for adjustment, as marked in Figure 6a. The first experiment considered only single-position mass change, and 15 structural local mass change patterns (from Scenario 1 to Scenario 15) were designed, with Scenario 0 being the scenario of no structural change. The second experiment considered the scenario of simultaneous application of additional mass at two locations. Ten multi-parameter variation scenarios were designed. The specific test scenarios were as shown in Table 2 and Table 3, and each scenario was tested by means of a 600 s random load excitation test, and the vibration signals of different scenario accelerometer combinations were collected using the dynamic signal acquisition system with a sampling frequency of 1000 Hz. The data obtained in the experimental processes from Scenario 0 to Scenario 15 were grouped into the first dataset, named Dataset I, and the data obtained in the experimental processes from Scenario 16 to Scenario 25 were grouped into the second dataset, named Dataset II.

### 4.2. Data Pre-Processing

Before training the 1D-CNN model, the complete dataset collected from the experiment needed to be preprocessed. Firstly, the data were normalized to the same scale using the method described in Section 3.1, and the normalized data were enhanced using the sliding window technique, when a sliding window size of 1000 × 7 was employed, with a stride of 500, and the data of 600 s collected from each scenario were divided into 1199 samples. Two-thirds of the data samples were used for model optimization and training, and the remaining one-third of the data samples were used for cross-validation of the model performance. When the model was trained iteratively, the input dataset was often processed into batches, where the minimum batch size was set to 64, and each training was randomly mixed and washed in batch order according to the law of uniform distribution, with the aim of ensuring that all signals are used with the same probability for training and testing.

### 4.3. Hyperparameter Optimization

**Figure 7:**
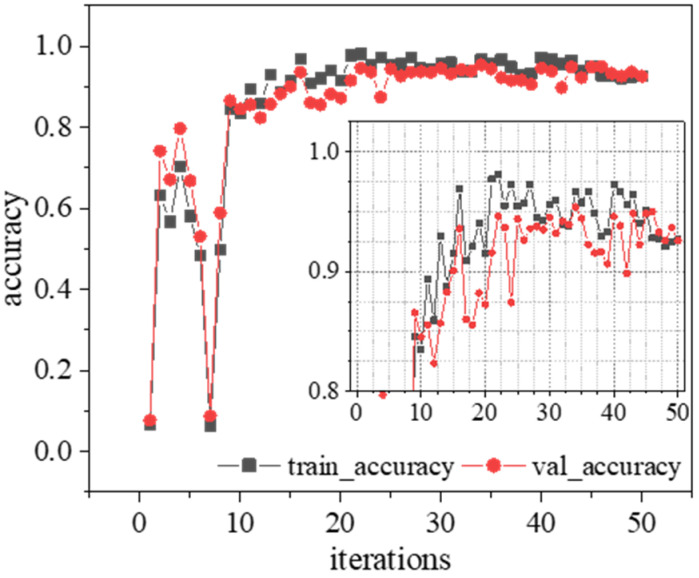
Network hyperparameter optimization process for identifying changes in simply supported beam architectures.

Dataset I was inputted into the 1D-CNN model architecture embedded with the Bayesian optimization algorithm built in Section 3, and the maximum number of iterations was set to 50. The accuracy of the hyperparameter optimization process of the Bayesian optimizer is shown in Figure 7. The runtime was obtained by measuring the execution time of the algorithm from the initial start of the algorithm to the result consisting of the best network found. The runtime in this processing was 9 h, 13 min and 36 s. The model training process mainly occupied GPU memory, and used all of the available GPU memory. The optimal result was the hyperparameter combination obtained at the 34th iteration, with a training accuracy of 99.061% when trained for 200 epochs. The test set test accuracy was 96.810%, and the configuration of the 1D-CNN architecture referenced for identifying structural changes in simply supported beams is shown in Table 4.

### 4.4. Analysis of Decision Fusion Identification Results

Based on the optimal combination of hyperparameters, the decision level data fusion method introduced in Section 3.4 was adopted. The optimized 1D-CNN model and data fusion method were combined to build a comprehensive decision model for structural damage identification. This model was used to identify and analyze the single-parameter variation and multi-parameter variation in simply supported beam structures.

Firstly, to verify the robustness of this method in identifying local and single parameter changes in the structure, one-third of Dataset I was divided into 1280 validation samples using the sliding window technique in Section 3.1. The constructed synthetic decision model was used to identify the corresponding structural states of these verification set samples, achieving 100% accuracy in identifying the structural state mapped by each validation sample.

**Figure 8:**
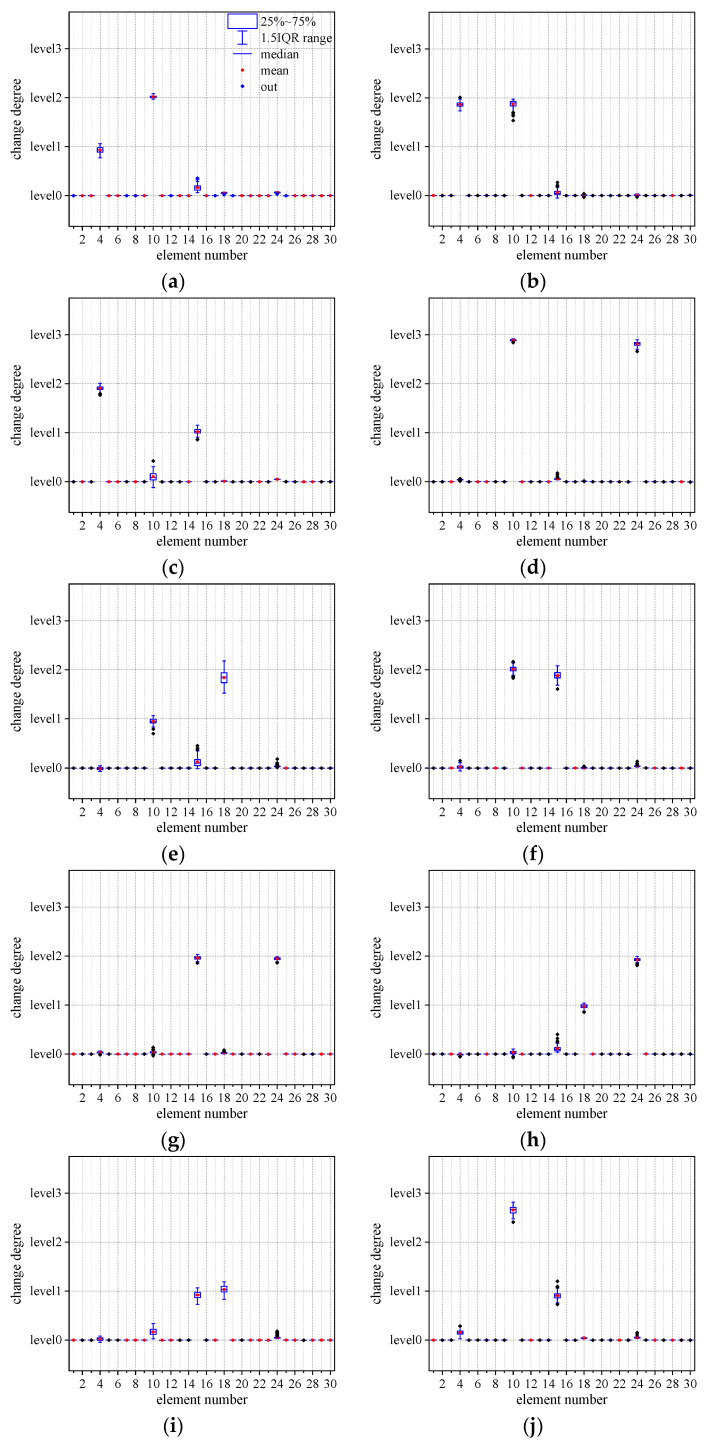
Statistics of identification results for the degree of structural change. (**a**) Scenario 16 identification results; (**b**) Scenario 17 identification results; (**c**) Scenario 18 identification results; (**d**) Scenario 19 identification results; (**e**) Scenario 20 identification results; (**f**) Scenario 21 identification results; (**g**) Scenario 22 identification results; (**h**) Scenario 23 identification results; (**i**) Scenario 24 identification results; (**j**) Scenario 25 identification results.

Next, the identification ability of this method for multi-position and multi-degree structural parameter changes was analyzed. For the scenarios ranging from Scenario 16 to Scenario 25, each scenario had 80 validation samples. The identification results were statistically analyzed by box plot, as shown in Figure 8. The damage location combinations for each scenario could be accurately identified and located, and the regression predictions for the degree change also showed a relatively high level of accuracy. There were certain deviations in the identification accuracy of the degree of changes in the sample in Scenario 20 and Scenario 25. Among the identification results of Scenario 20, the prediction results of the change degree of element 15 showed instability, and the deviation range from the actual degree was relatively large. In the identification results of Scenario 25, there was a certain deviation in the prediction results of the degree of change in element 10. The mean value of all of the prediction results deviated from the actual degree value, but still tended towards the actual degree value. Overall, this method has excellent identification ability for local position changes in simply supported beam structures.

## 5. Case Study 2: Identification of Frame Structure Joint Damage

### 5.1. Introduction to the Model Test of the Frame Structure

**Figure 9:**
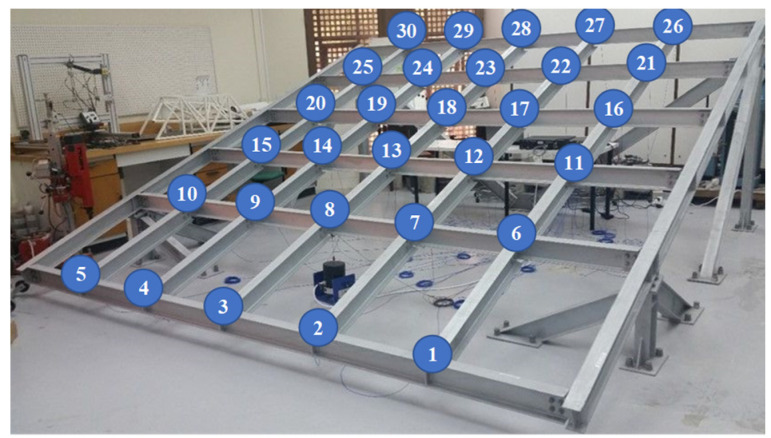
Simulation model of stadium stands [35].

To verify the effectiveness and generality of the proposed method for different structural damage detection problems, a large frame test structure, designed and built in the structural laboratory of Qatar University, was selected as the research object; the structure mainly consisted of eight main beams and 25 support elements, which were connected by 42 bolts. The mechanism simulated damage to the frame structure by releasing the nodal bolts. The experiment included 31 test conditions, including a healthy condition and 30 damage conditions, corresponding to 30 single nodal damage instances (the damage locations were numbered corresponding to the nodal locations in Figure 9). Two experiments were conducted for each test condition using random vibration generated by the exciter as the excitation source. To collect structural acceleration data, 30 acceleration sensors were installed at the nodes at which the 30 damage locations were set in the frame structure (as indicated by the numbered positions in Figure 9). Two experiments were conducted, and each lasted for 256 s, with a sampling frequency of 1024 Hz. The signal length recorded by each sensor was 262,144 data joints. Therefore, two datasets (Dataset A and Dataset B) were created, with each containing 8,126,464 data joints. For more information about the experimental datasets, the reader is referred to the following website: http://www.structuralvibration.com/benchmark/ (accessed on 4 January 2023).

### 5.2. Model Optimization and Analysis of Identification Results

**Figure 10:**
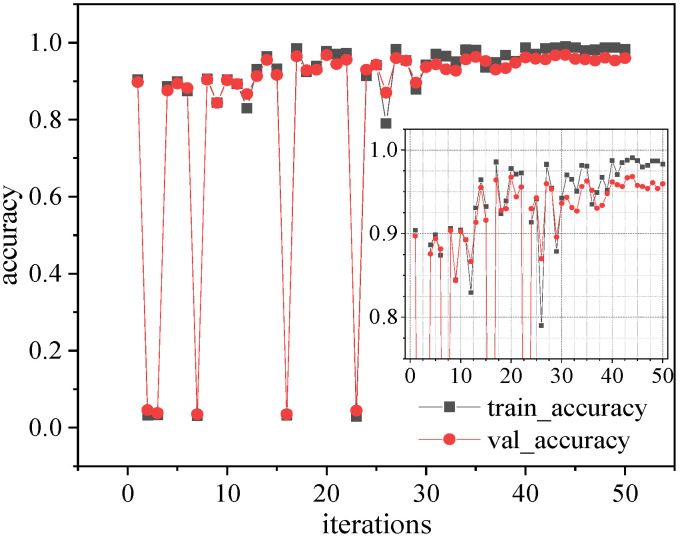
Hyperparameter optimization process for identifying damage to frame structures.

Dataset A was used to perform hyperparameter optimization, training and testing for the 1D-CNN model, and the training and testing sets were established using the data preprocessing method described in Section 3.1. The dimensions of each sample were 1024 × 12, where the signals from 12 sensors (The serial number of the 12 sensors are respectively 2, 3, 4, 7, 8, 9, 12, 13, 14, 22, 23, 24, 27, 28, 29) were used. A discussion of the sensor arrangement can be found in Section 6.1. The 1D-CNN architecture with embedded Bayesian optimization algorithm was trained using the training and test sets, and the Bayesian optimization hyperparameter process was performed for 50 iterations, with 200 epochs set for each iteration. The value of batch size was taken to be 64. In the model optimization process, the runtime for this processing was 13 h, 35 min and 27 s, The model training process mainly occupied GPU memory, and used all of the available GPU memory. The results of the hyperparameter optimization process are shown in Figure 10, and the model exhibited the best performance at the 44th iteration. The specific hyperparameter combinations are shown in Table 5.

During the identification stage, the decision layer fusion strategy proposed in this paper was used for the identification of frame structure node damage based on the optimized 1D-CNN model. Dataset B was used as the validation set, and only the data from 12 sensors were used. The accuracy of damage identification for joint damage reached 99.85% using this method. In order to highlight the advantages of the proposed damage identification strategy, a comparison was performed with other studies utilizing the same public dataset for deep learning damage identification research. As shown in Table 6, the method proposed in this paper achieved the highest average accuracy for joint damage identification in the frame structure, when compared to the four methods described in four other studies. The method combining the continuous wavelet transform method with the 2D-CNN model also demonstrates a relatively high identification accuracy that is comparable to the method proposed in this paper. However, tuning problems such as the selection of wavelet basis functions when converting acceleration signals to two-dimensional time–frequency spectrograms using the continuous wavelet transform method are very complicated, and this method requires a relatively high number of training samples and computational requirements. It is worth noting that only the data from 12 sparsely distributed sensors were used in the model training process in this paper. This has important engineering significance for optimizing the structural health monitoring perception network, and may also provide some reference values for the practical application of deep learning-based structural damage identification methods in the future.

## 6. Discussion

In the previous sections, it was shown that the proposed method has strong robustness in application on two structures. In this section, the generalization ability of the proposed method to different degrees of data quality will be discussed. Firstly, the sensitivity analysis of the four main parameters, including the number and location of sensors and measurement points, signal noise, input data size, and batch size were conducted for the proposed method. Finally, the scalability of the proposed method to datasets of different scales was verified by establishing datasets with different scales through resampling.

### 6.1. Influence of Sensor Arrangement

In the field of structural health monitoring, the arrangement of sensor measurement points is an extremely important aspect that can affect the results of the corresponding study. Based on the vibration-based structural damage identification method, accelerometer arrangement will affect the analysis of the overall dynamic characteristics of the structure, as shown in Equation (8) for the dynamic equations of the structure.

(8) $$\left[M_{b}\right]\left\{{\overset{\cdot \cdot }{Z}}_{b}\right\}+\left[C_{b}\right]\left\{{\overset{\cdot }{Z}}_{b}\right\}+\left[K_{b}\right]\left\{{\overset{}{Z}}_{b}\right\}=\left\{F_{b}\right\}$$

where $\left[M_{b}\right],\left[C_{b}\right],\left[K_{b}\right]$ are the mass matrix, damping matrix and stiffness matrix of the structure, respectively, $\left[F_{b}\right]$ is the external excitation matrix of the structure, and $\left[Z_{b}\right]$ is the displacement matrix of the structure. The vibration form φ of the free vibration of the structure has orthogonality, and the characteristic equation of the above vibration equation can be rewritten as:

(9) $$\left[K_{b}\right]\varphi =\omega^{2}\left[M_{b}\right]\varphi$$

**Figure 11:**
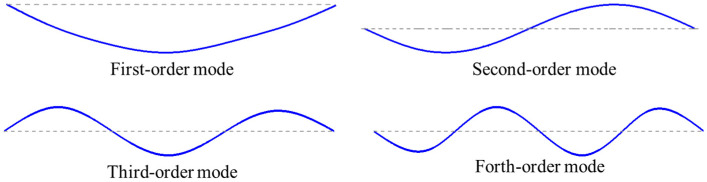
Diagram of the first four orders of vibration in the simply supported beam.

As can be seen from the above equation, when the stiffness or mass of the structure changes, the inherent frequency and vibration pattern will change to a certain extent. When the special measurement point location of the structure is not considered, the sensor should be arranged at a location that is sensitive to the vibration pattern and frequency change as much as possible. For the simply supported beam structure, its first four orders of inherent frequency were 9.77 Hz, 37.11 Hz, 82.0313 Hz, 152.34 Hz, corresponding to the vibration pattern shown in Figure 11; in this study, seven locations were selected as measurement points for the first four orders of vibration peaks, corresponding to the location, and with an equal number of uniform arrangement schemes, with different numbers of sensors in each scheme. For comparison, the schemes are presented in Table 7, where the specific sensor locations are described by the distance of the sensor from the left-end support. Using the optimal network model designed and tested in Section 3.2, the results show that the measurement points at the locations corresponding to the peaks are most favorable for the identification of structural parameter changes; the accuracy rates of scheme 1 and scheme 2 in Table 7 are higher, and the measurement point arrangement form of scheme 4 in Table 7 is not able to express the overall dynamic characteristics of the structure. Therefore, when using sparse sensor arrangements, positions corresponding to vibration peaks should be given priority, which is more conducive to this method obtaining a higher identification accuracy.

**Figure 12:**
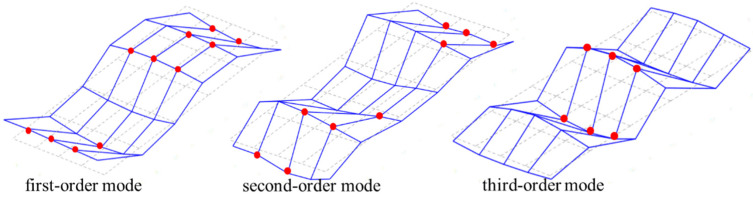
The first three orders of the vertical vibration pattern of the frame structure.

For the frame structure, when 30 nodes are used as sensor measurement points, the signal data from 30 sensors are used to train the proposed 1D-CNN model, and the model is not able to converge. The reason for the analysis is that there is too little damage information in the data sample, which is covered and diluted by a large number of non-damage information data. Specifically, among the 30 pieces of signal data, only one piece of signal data comes from the accelerometer at the location of bolt loosening, and the other 29 signal data come from accelerometers at the location of nodes in health states, so it is difficult to extract the damage characteristics from these numerous data. Therefore, a sparse sensor layout is selected in this paper for performing the overall detection of the frame structure. The first three order modes of the frame structure were obtained using the stochastic subspace identification method, as shown in Figure 12, and the node locations with relative amplitude of modes greater than 0.8 are marked in red. After comprehensive analysis of the first three vertical order modes, four relatively optimal sensor layout schemes were selected, as shown in Table 8. After testing, the dataset collected by the sensor measurement points in scheme 2 was found to be more suitable for the training and verification of the proposed model.

Through two examples, it was found that for the 1D-CNN model proposed in this paper, the datasets collected by the sensors of different arrangement forms affect the identification accuracy of the network model. It was verified on the basis of several experiments that the position corresponding to the peak of the structural vibration modes had been used as the measurement point. The probability of obtaining a good dataset is higher, such that the 1D-CNN model can identify local parameter changes in a structure with higher accuracy.

### 6.2. Sensitivity Analysis for Input Data Size and Batch Size

**Figure 13:**
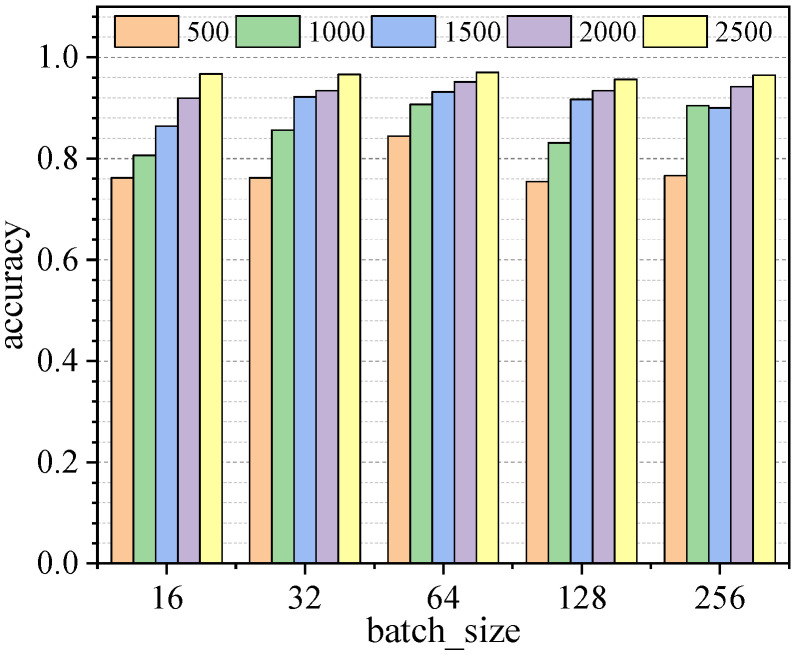
Accuracy statistics of identification results.

**Figure A1:**
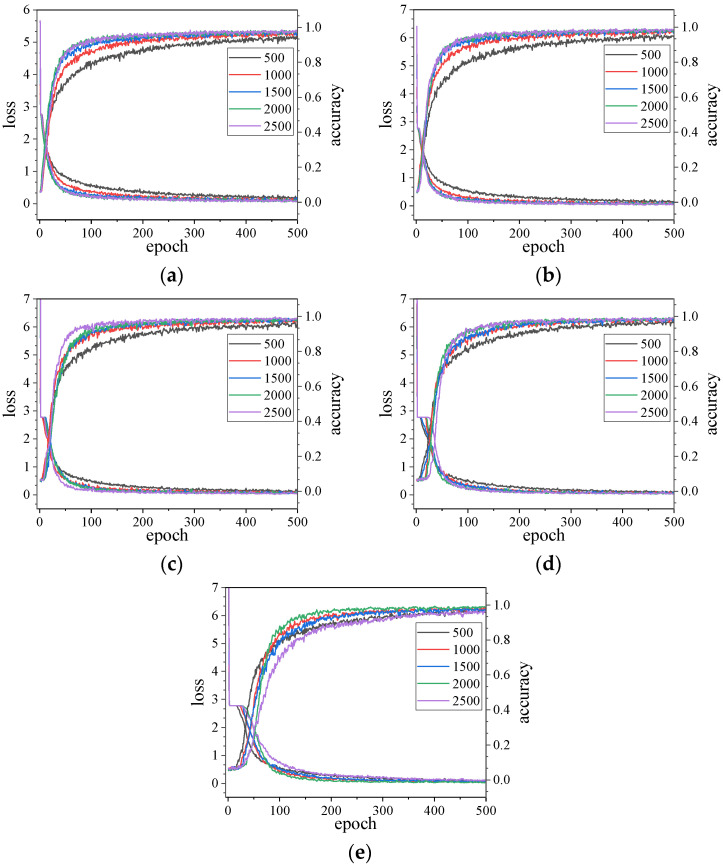
Training process on the dataset of simply supported beams with different input data sizes. (**a**) Batch size = 16; (**b**) Batch size = 32; (**c**) Batch size = 64; (**d**) Batch size = 128; (**e**) Batch size = 256.

**Figure A2:**
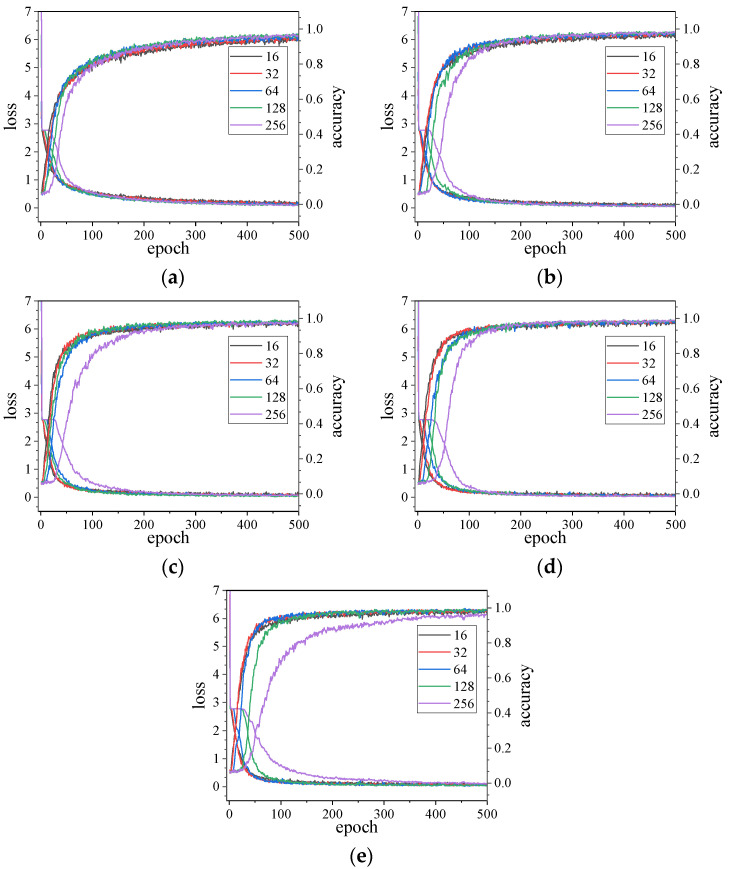
Training process on the dataset of simply supported beams with different batch sizes. (**a**) Input data size = 500; (**b**) Input data size = 1000; (**c**) Input data size = 1500; (**d**) Input data size = 2000; (**e**) Input data size = 2500.

Some of the training hyperparameters are not suitable for calculation by the Bayesian optimization algorithm, and as is the case for the input data size and batch size of the 1D-CNN model, they are important for the results of model training; therefore, a comparison experiment was designed to analyze the sensitivity of the two parameters on the identification accuracy. The h values of input data size were taken as 500, 1000, 1500, 2000, 2500, and the batch size values were taken as 16, 32, 64, 128, 256, respectively; 500 epochs of training were performed, and the accuracy and loss values of the training process are shown in Appendix A Figure A1 and Figure A2. The larger the h value is, the more information the input data sample contains, and the better the model training effect tends to be, where the difference between 2000 and 2500 values is not obvious, and tends to be optimal; batch size has a lower impact on the accuracy effect, but has a more obvious impact on the model convergence speed. Using 25 combinations of the above two parameters, a total of 25 optimal models were trained, and all the models were verified using Dataset I of the simply supported beam, and the accuracy of the identification results is shown in Figure 13. By comparison, it was found that when the h value of the input data was 2500 and the batch size was 64, the identification accuracy was the highest, at 97.03%.

**Figure 14:**
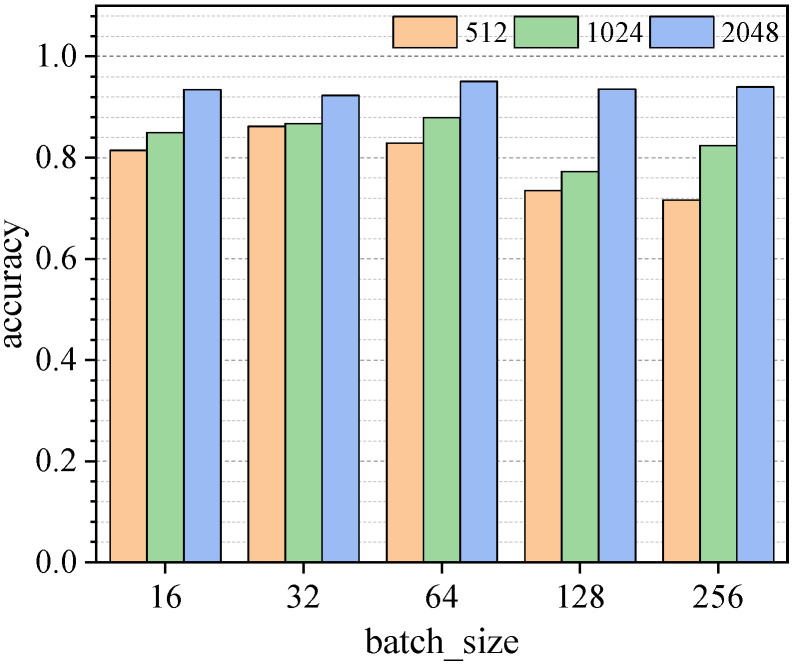
Accuracy statistics of identification results.

**Figure A3:**
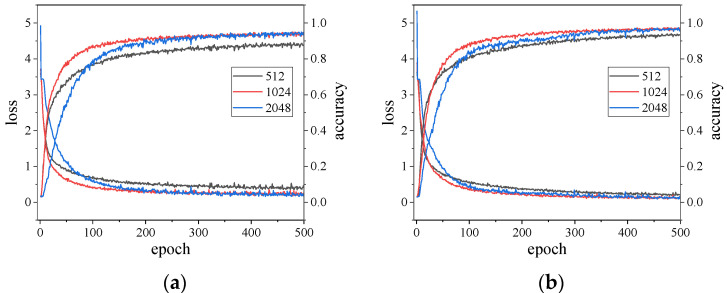

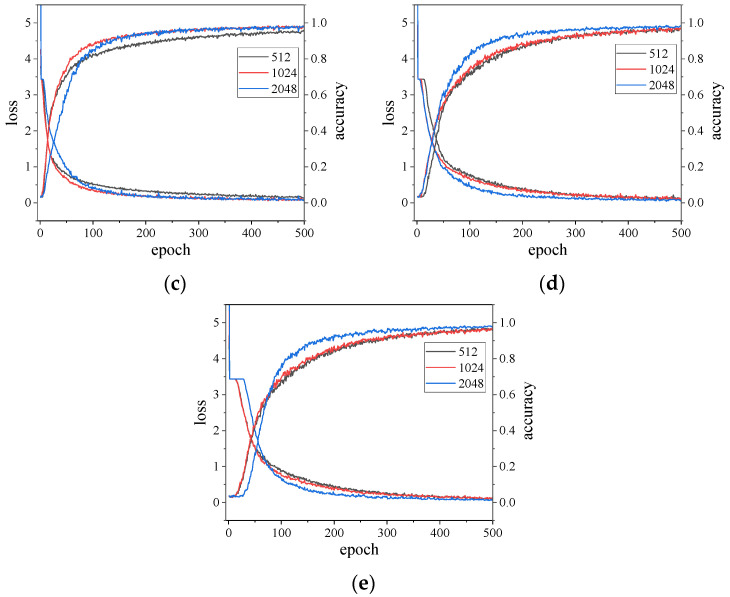
Training process on the frame structure dataset with different batch sizes. (**a**) Batch size = 16; (**b**) Batch size = 32; (**c**) Batch size = 64; (**d**) Batch size = 128; (**e**) Batch size = 256.

**Figure A4:**
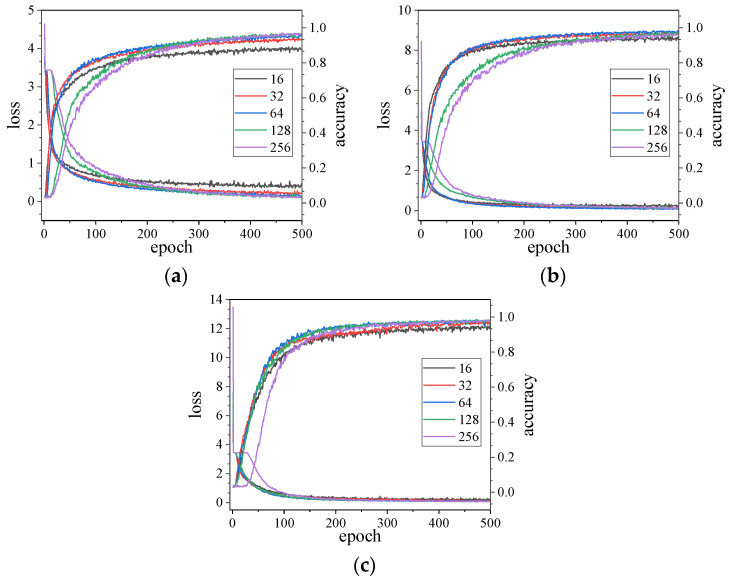
Training process on the frame structure dataset with different batch sizes. (**a**) Input data size = 512; (**b**) Input data size = 1024; (**c**) Input data size = 2048.

With the aim of obtaining the best combination of hyperparameters to identify the structural damage of the frame, the sensitivity of input data size and batch size to the results of model training was further discussed. The h values of the input data were 512, 1024, 2048, the batch size values were 16, 32, 64, 128, 256, and training was carried out for 500 epochs. The training process accuracy and loss values are shown in Appendix A Figure A3 and Figure A4. Dataset B was used for validation, and a comparison of the results is shown in Figure 14. The final determination of the selection of the h value was 2048, and the batch size value was 64; the identification accuracy of the validation set reached 95.06%.

### 6.3. Noise Resistance

In practical applications, environmental noise inevitably affects the quality of the monitored vibration signal, and the form of noise varies in different environments. To test the ability of the method proposed in this paper to resist noise, a method to reduce the signal-to-noise ratio (SNR) of the signal is used to simulate the effect of noise on the signal. The SNR was calculated as shown in Equation (10) and as shown in Equation (11). Different levels of noise were added to the acceleration signal of the validation dataset. In this way, the SNR of the signal in the validation dataset was reduced, and the accuracy of the damage identification of the model was verified by the dataset with noise.

**Figure 15:**
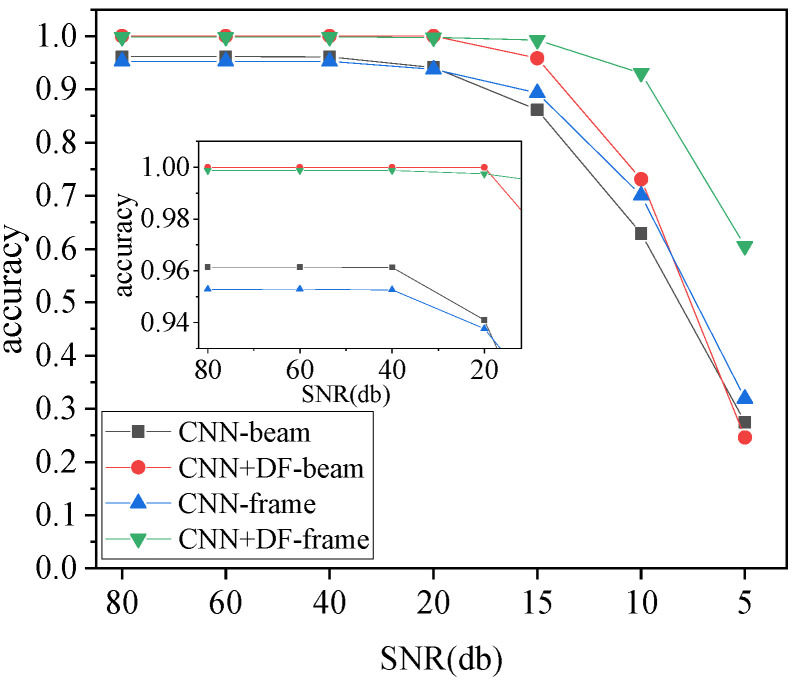
Impact of noise on identification results.

(10) SNR=10log10PsignalPnoise

(11) $$\hat{S_{n}}=S_{n}+\xi$$

(12) $$\xi ∼N\left(0,\sigma^{2}\right)$$

where $S_{n}$ is the signal without noise addition, $\hat{S_{n}}$ is the signal after noise addition, and *ξ* is a random variable following a Gaussian distribution with mean 0 and variance $\sigma^{2}$. In this work, the σ values of the signals with different SNRs that require noise addition were calculated by Equation (10). Seven SNRs values—80 dB, 60 dB, 40 dB, 20 dB, 15 dB, 10 dB, and 5 dB—were selected to process the verification sets of the simply supported beam case and the frame structure case. Respectively, these datasets were identified using the optimized model, and the damage identification accuracy is shown in Figure 15.

The results of the two case studies showed that the optimized 1D-CNN model maintained good identification accuracy when the SNR was greater than 40 dB. When the SNR decreased to the range of 40 dB to 20 dB, the identification accuracy of the 1D-CNN model for structural parameter changes in both cases showed a downward trend. When the SNR was 20 dB, the identification accuracy of the 1D-CNN model for structural changes in simply supported beams decreased to 94.1%, and the identification accuracy of joint damage to the frame structure decreased to 93.76%. The damage identification method proposed in this paper, which combines 1D-CNN and data fusion technology, maintained high accuracies of 100% and 99.75% for the identification of simply supported beam structural signals and frame structural signals with an SNR of 20 dB. When the SNR was lower than 20 dB, the identification accuracy of the 1D-CNN model decreased significantly. The identification accuracy of the structural parameter changes in the simply supported beam decreased to 86.15% when the SNR was 15 dB and to 62.9% when the SNR was 10 dB. The identification accuracy of the frame structure node damage decreased to 89.34% when the SNR was 15 dB and to 70.06% when the SNR was 10 dB. The comprehensive decision model built by data fusion technology was used to identify signals within an SNR range of 40 dB to 20 dB. The identification results of structural damage in two cases still maintained accuracy of over 95%, but when the SNR was 10 dB, the identification accuracy of the structural parameter changes in the simply supported beam decreased to 73.13%. The main reason for this is that noise reduces the identification ability of the model to the degree of structural parameter change. Although data fusion technology has greatly improved the identification ability of the 1D-CNN model, when the identification ability of the 1D-CNN model is poor, the improvement effect of data fusion technology on its ability is no longer obvious. Therefore, this paper first adopted the Bayesian optimization algorithm to seek the hyperparameters of the 1D-CNN model with high identification accuracy, and then applied data fusion technology. This not only greatly improved the model identification ability, it also showed strong anti-noise ability.

### 6.4. Identification Result on Extended Dataset with Different Scales

In order to further verify the effectiveness of the proposed method, we applied the optimized model and decision fusion strategy proposed in this paper to datasets of different scales. In the case of simply supported beams, 200 s data for each scenario from Dataset I were selected as the validation set, and six validation sets with six different scales were established. The number of samples contained in each dataset was 1600, 3200, 8000, 16,000, 32,000, and 80,000, respectively. Taking the dataset with 1600 samples as an example, 100 random numbers were selected from 200,000 data points in each scenario as the starting point index for each sample. Each sample was sampled with 2500 data points. Using this method, 100 samples were generated for each scenario, and a dataset with 1600 samples was established for 16 single-parameter variation scenarios of the simply supported beam. For the frame structure case, 256 s data of each working condition from Dataset B were selected as the validation set, and six validation sets with six different scales were established. The number of samples contained in each dataset was 3968, 7936, 15,872, 31,744, 63,488, and 126,976, respectively. As shown in Table 9 and Table 10, on all extended datasets, the validation accuracy of the method proposed in this paper exceeded 99%, and the runtime during the identification process was far lower than the vibration signal acquisition time, making real-time diagnosis of structural damage possible. Overall, the method proposed in this paper has good scalability on datasets of different scales.

## 7. Conclusions

For structural dynamic properties, the effects of different structural forms and different structural damages are very complex. Therefore, it is not easy to adjust the hyperparameters of the 1D-CNN model to suit specific structural damage diagnosis problems. In this paper, we proposed embedding a Bayesian optimization algorithm into the 1D-CNN model in order to adapt the hyperparameters of the model to specific problems. In addition, this paper also considered the application of the decision-level fusion technique to the model damage diagnosis stage to solve the problem of misclassification due to the poor quality of data samples. In the self-designed simple-supported beam test, the proposed method was able to identify the changes in the local location of the structure with 100% accuracy, and predict the degree of structural parameter changes quite accurately. In addition, publicly available datasets were used for validation. Twelve sparsely arranged sensor data were utilized, and 30 loosened node bolts could be identified with an accuracy of 99.84%. Finally, the influence of sensor layout and noise on our proposed method was discussed. It was verified that the node locations with peak value in the structural vibration mode are suitable for placing sensors, because the signals collected at these locations were more sensitive to structural changes, and the sparse sensor array was able to meet the monitoring requirements of the whole structure. In addition, the method exhibited strong immunity to noise, and the identification results of the simple beam structure verification set and the frame structure dataset still maintained 100% and 99.75% accuracy after adding white noise to the original signal with an SNR as low as 20 dB. Furthermore, the proposed method has good scalability for datasets of different sizes.

Before applying the proposed method to real engineering structures, it is necessary to obtain a large number of data samples related to actual engineering, containing various different labels. However, this is obviously a difficult if not impossible problem to solve. Therefore, in future work, a combination of model-driven and data-driven approaches will be considered to reduce the discrepancy between numerical models and actual engineering by generating a certain number of data samples with labels through numerical simulation techniques for training damage diagnosis models. Additionally, we will consider the use of migration learning methods to solve the problem of the small number of data samples.

## Figures and Tables

**Table 1:** Hyperparameter range.

Block	Hyperparameter Name	Range to Probe
	Number of Conv_Block	1 to 5
The first Conv_Block	Number of layers	1, 2
	Number of filters	8, 16
	Kernel size	20 to 100
	Padding	valid, same
	Activation	ReLU, Tanh, Leaky ReLu
Other Conv_Blocks	Number of layers	1, 2
	Number of filters	32 to 200
	Kernel size	3 to 20
	Padding	valid, same
	Activation	ReLU, Tanh, Leaky ReLu
Classifier block	Number of FC layers	2 to 5
	Number of nodes in FC hidden layers	64 to 1024
	Dropout ratio	0, 0.1, 0.2, 0.3, 0.4, 0.5
	Training optimizers	SGD, RMSprop, Adam

**Table 2:** Description of a single change scenario of a simply supported beam structure.

Change Level	4# Element	10# Element	15# Element	18# Element	24# Element
Level 1	Scenario 1	Scenario 4	Scenario 7	Scenario 10	Scenario 13
Level 2	Scenario 2	Scenario 5	Scenario 8	Scenario 11	Scenario 14
Level 3	Scenario 3	Scenario 6	Scenario 9	Scenario 12	Scenario 15

**Table 3:** Description of multi-parameter variation scenarios for simply supported beam structures.

Scene	Description of Changes	Scene	Description of Changes
Scenario 16	4# (Level 1) + 10# (Level 2)	Scenario 21	15# (Level 2) + 10# (Level 2)
Scenario 17	4# (Level 2) + 10# (Level 2)	Scenario 22	24# (Level 2) + 15# (Level 2)
Scenario 18	4# (Level 2) + 15# (Level 1)	Scenario 23	24# (Level 2) + 18# (Level 1)
Scenario 19	24# (Level 3) + 10# (Level 3)	Scenario 24	15# (Level 1) + 18# (Level 1)
Scenario 20	10# (Level 1) + 18# (Level 2)	Scenario 25	10# (Level 3) + 15# (Level 1)

**Table 4:** Network structure for identifying changes in the architecture of simply supported beams.

Block	Type	Kernel (Node) Number	Kernel Size	Stride	Padding	Activation
Block 1	Conv1D	16	43	1	same	ReLU
	Batch Normalization	-	-	-	-	-
	Maxpool1D	-	2	1	-	-
Block 2	Conv1D	32	13	1	same	ReLU
	Batch Normalization	-	-	-	-	-
	Maxpool1D	-	2	1	-	-
Block 3	Conv1D	64	7	1	same	ReLU
	Batch Normalization	-	-	-	-	-
	Maxpool1D	-	2	1	-	-
	Flatten	-	-	-	-	-
Classifier block	Dense	256	-	-	-	-
	Dropout	-	-	-	-	-
	SoftMax	-	-	-	-	-

**Table 5:** Network-optimized parameter combinations for identifying damage to frame structures.

Block	Type	Kernel (Node) Number	Kernel Size	Stride	Padding	Activation
Block 1	Conv1D	16	88	1	valid	ReLU
	Batch Normalization	-	-	-	-	-
	Maxpool1D	-	2	1	-	-
Block 2	Conv1D	64	13	1	valid	ReLU
	Batch Normalization	-	-	-	-	-
	Maxpool1D	-	2	1	-	-
Block 3	Conv1D	128	10	1	valid	ReLU
	Batch Normalization	-	-	-	-	-
	Maxpool1D	-	2	1	-	-
	Flatten	-	-	-	-	-
Classifier block	Dense	256	-	-	-	-
	Dropout	-	-	-	-	-
	Softmax	-	-	-	-	-

**Table 6:** Comparison of QUGS structural damage identification methods based on deep learning.

Literature	Model	Sensor Numbers	Detection Range	Mean Accuracy
Chen et al. [32]	2D-CNN + CWT	30	Whole structure	99.61%
Flah et al. [44]	1D-CNN	30	Whole structure	86%
Truong et al. [49]	1D-CNN + GRU	1	One joint	91.31%
Azimi et al. [50]	extremely compressed data + 1D-CNN	30	Whole structure	91.9%
Proposed method	1D-CNN + DF	12	Whole structure	99.85%

**Table 7:** Sensor measurement point arrangement.

Scheme	Number of Sensors	Specific Sensor Location	Accuracy
scheme 1	5	0.25, 0.375, 0.75, 1.125, 1.25	95.18%
scheme 2	7	0.25, 0.375, 0.55, 0.75, 0.95, 1.125, 1.25	97.03%
scheme 3	7	0.2, 0.375, 0.55, 0.75, 0.95, 1.125, 1.3	87.17%
scheme 4	4	0.25, 0.375, 0.55, 0.75	63.28%

**Table 8:** Sensor measurement point arrangement.

Scheme	Number of Sensors	Specific Sensor Location	Accuracy
scheme 1	10	6#–7#–8#–9#–10#–21#–22#–23#–24#–25#	93.15%
scheme 2	12	2#–3#–4#–7#–8#–9#–12#–13#–14#–22#–23#–24#–27#–28#–29#	95.06%
scheme 3	15	6#–7#–8#–9#–10#–16#–17#–18#–19#–20#–26#–27#–28#–29#–30#	89.44%
scheme 4	18	2#–3#–4#–7#–8#–9#–12#–13#–14#–17#–18#–19#–22#–23#–24#–27#–28#–29#	90.57%

**Table 9:** Identification results on extended datasets of the simply supported beam with different scales.

Dataset Capacity	Accuracy	Runtime	Signal Acquisition Time
1600	100%	9.54 s	12,000 s
3200	100%	22.39 s	24,000 s
8000	100%	49.36 s	60,000 s
16,000	99.994%	101.11 s	120,000 s
32,000	99.994%	222.96 s	240,000 s
80,000	99.993%	661.50 s	600,000 s

**Table 10:** Identification result on extended datasets of the frame structure with different scales.

Dataset Capacity	Accuracy	Runtime	Signal Acquisition Time
3968	99.849%	29.37 s	23,808 s
7936	99.811%	54.60 s	47,616 s
15,872	99.836%	98.91 s	95,232 s
31,744	99.817%	239.90 s	190,464 s
63,488	99.735%	506.34 s	380,928 s
126,976	99.746%	1062.43 s	761,856 s

## Data Availability

Restrictions apply to the availability of these data. Data were obtained from Onur AVCI’s team and are available from the at http://www.structuralvibration.com/benchmark/ (accessed on 4 January 2023) with the permission of Onur AVCI’s team.
